# The Medical Association of Georgia

**Published:** 1887-04

**Authors:** 


THE MEDICAL ASSOCIATION OF GEORGIA meets in Atlanta on the third Wednesday in the present month. A large attendance is expected, and many interesting matters will come before the body. We are not prepared at this writing to state all that will be done to make the occasion an agreeable and profitable one to the brethren of the profession, who will do themselves and the citizens of Atlanta the pleasure of attending this meeting. Among other things, we will mention that a sumptuous banquent will be given the members of the Association at the Kimball House on Thursday night, the 21st; there will also be given a free excursion, over the Georgia Pacific Railroad, to Lithia or Salt Spring, twenty miles West of Atlanta. For the latter we shall be indebted to the liberality of our most estimable fellow-citizen, G. W. Marsh, Esq., of the firm of Moore, Marsh & Co.

